# Multidisciplinary respiratory rehabilitation in combination with non-invasive positive pressure ventilation in the treatment of elderly patients with severe chronic obstructive pulmonary disease

**DOI:** 10.12669/pjms.35.2.459

**Published:** 2019

**Authors:** Limin Cui, Haixia Liu, Lei Sun

**Affiliations:** 1 *Limin Cui Binzhou People’s Hospital, Shandong, 256610, China*; 2 *Haixia Liu Binzhou People’s Hospital, Shandong, 256610, China*; 3 *Lei Sun Binzhou People’s Hospital, Shandong, 256610, China*

**Keywords:** Chronic obstructive pulmonary disease, Pulmonary rehabilitation, Non-invasive positive pressure ventilation

## Abstract

**Objective:**

To explore the effectiveness of multidisciplinary comprehensive respiratory rehabilitation in combination with non-invasive positive pressure ventilation (NIPPV) in the treatment of elderly patients with severe chronic obstructive pulmonary disease (COPD).

**Methods:**

Eighty-eight elderly patients with severe COPD who were admitted by the hospital between February 2016 and April 2017 were enrolled and grouped into a control group (n=29), intervention Group-A (n=30) and intervention Group-B (n=29) according to random sampling. Patients in the control group were given medicines and oxygen therapy; intervention Group-A was given NIPVV in addition to medicines and oxygen therapy; intervention Group-B was given multidisciplinary comprehensive respiratory rehabilitation in addition to the treatment as the intervention Group-A. Cardiopulmonary exercise testing, body mass index, BODE index score (airflow obstruction, dyspnea, and exercise capacity index), scoring of quality of life and arterial blood gas analysis were performed before treatment and in the 3^rd^ month after treatment.

**Results:**

The maximum exercise power (W_max_), maximum oxygen uptake (VO_2max_), six minutes walking distance (6MWD), modified British Medical Research Council (MMRC), BODE index, score of quality of life, arterial partial pressure of carbon dioxide (PaCO_2_) and arterial partial pressure of oxygen (PaO_2_) of intervention Group-A and b were significantly improved after treatment (P<0.05); the differences with the control group had statistical significance (P<0.05). The improvement of 6MWD, MMRC, score of quality of life, PaO_2_ and PaCO_2_ of intervention Group-B was superior to that of intervention Group-A. (P<0.05).

**Conclusion:**

Multidisciplinary comprehensive respiratory rehabilitation in combination with NIPPV can further relieve dyspnea of patients, enhance exercise tolerance and quality of life, and facilitate recovery; hence it is worth application and promotion.

## INTRODUCTION

Chronic Obstructive Pulmonary Disease (COPD), a common and frequently-occurring disease in respiratory system, is mainly featured by continuous airflow limitation and manifests as cough and wheeze.^1^,^2^ Disorder of ventilation and gas exchange may occur to patients with COPD, which can limit their exercise capacity.^3^ COPD a high fatality rate. After the acute stage, the symptoms can be relieved, but pulmonary function is still getting worse; hence it is not beneficial to defend external adverse factors, and repeated attack is easy to induce various complications.^4^ COPD has a high incidence among middle-aged and elderly people. A recent investigation has suggested that the incidence of COPD among elderly in China tends to be increase year by year with the acceleration of aging tendency,^5^ which greatly increases social and economical burdens and has been a serious public problem.

Respiratory rehabilitation, i.e. pulmonary rehabilitation, refers to multidisciplinary comprehensive intervention measures given to patients with chronic respiratory disease who have symptoms and weakened activity of daily living, which has been an indispensable part in the standard treatment of COPD. It can relieve respiratory symptoms, increase exercise tolerance and improve living quality of patients with COPD.^6^,^7^ The Global Initiative for Chronic Obstructive Lung Disease (2006) points out that pulmonary rehabilitation treatment should be performed along with conventional drug treatment in the treatment of patients with moderate and severe COPD. Effective comprehensive respiratory rehabilitation in combination with non-invasive positive pressure ventilation (NIPPV) is an effective therapy for relieving dyspnea and improving living quality of COPD patients. In the present study, 44 elderly patients with severe COPD were treated using multidisciplinary comprehensive respiratory rehabilitation in combination with NIPPV, and the therapy was found having favorable effect.

## METHODS

Eighty-eight elderly patients with severe COPD who were admitted into Binzhou People’s Hospital, Shandong, China, between February 2016 and April 2017 were enrolled. The inclusion criteria included having stable condition of COPD for at least two weeks, having severity of pulmonary function at grade III or IV, satisfying the diagnostic criteria described in the Guidelines for the Diagnosis and Treatment of COPD,^8^ and not having short-acting bronchodilators in 6 h before trial and long-acting bronchodilators in 24 h before trial. Those who had severe hepatic disease and renal disease, cognitive impairment, severe extremity function disturbance especially lower limbs function disorder, or other respiratory tract diseases were excluded. The included patients were divided into intervention Group-A (n=30), intervention Group-B (n=29) and control group (n=29). There were 42 cases of hypertension, 13 cases of coronary heart disease, 25 cases of Type-2 diabetes mellitus, 9 cases of bronchiectasia and 10 cases of obsolete pulmonary tuberculosis. The differences of gender, age, forced expiratory volume in one second (FEV1), forced vital capacity (FVC) and FEV1 predicted (FEV1%) between the two groups had no statistical significance (P>0.05) (Table-I). All the patients signed informed consent after detailed counselling, and the study protocol has been reviewed and approved by the medical ethics committee of our hospital.

**Table-I:** General data between the three groups.

Group	Control group	Intervention group 1	Intervention group 2
Gender (male/female)	17/12	16/14	18/11
Age (year)	67.84±5.53	68.46±5.39	68.67±5.42
FEV1(L)	1.16±0.16	1.16±0.10	1.15±0.13
FEV1%	42.26±5.12	42.02±4.53	43.24±4.94
FVC (L)	2.20±0.22	2.21±0.31	2.22±0.30
FEV1 /FVC(%)	52.76±4.84	52.93±5.22	52.55±6.15

Patients in both groups were given conventional therapies to relieve cough, resolve phlegm and dilate bronchus. Bronchodilators were limited to doxofylline tablets (0.2 g each time, twice a day), 500 μg of fluticasone propionate and 50 μg of salmeterol xinafoate and fluticasone propionate powder for inhalation (one inhalation each time, twice each day), and salbutamol sulphate aerosol (1 or 2 press each time, 100 μg each press). Patients with combination of diseases should be treated according to relevant guidelines.

Patients in the control group were given conventional drug treatment and oxygen therapy only. Time of oxygen inhalation via nasal tube was 10~15 h/day. Flow of oxygen was between 1.5 L/min and 2.5 L/min. Daily activity was maintained. They were reexamined after three months of treatment.

Patients in intervention group 1 were given NIPPV in addition to conventional medicine treatment and oxygen therapy. BIPAP-Synchrony non-invasive ventilator (Respironics Inc., USA) was used. Mode of ventilation was determined according to the categories of respiratory failure. Patients with Type-I respiratory failure were given continuous positive airway pressure (CPAP) mode, while patients with type II respiratory failure were given S/T mode. The initial parameter of CPAP mode was six cm H_2_O; as to S/T mode, inspiratory positive airway pressure was set as 8~12 cm H_2_O, and expiratory positive airway pressure was set as 4 cm H_2_O. Backup frequency was set as 15 times/min. Oxygen flow of the breathing machine was set as 3~6 L/minutes. The parameters of the breathing machine could be adjusted according to the changes of the disease condition. NIPPV lasted for at least 6 h each day. The treatment lasted for three months.

Patients in intervention group 2 were given multidisciplinary comprehensive respiratory rehabilitation in addition to conventional drug treatment, oxygen therapy and NIPPV. The treatment lasted for three months. Multidisciplinary comprehensive respiratory rehabilitation included health education, exercise training, medication guidance, nutrition guidance and social psychological support. As to health education, medical staffs from department of respiration informed the patients with knowledge of health at regular intervals. The major content included avoiding the pathogenic risk factors of COPD such as smoking and away from dust, smoke and harmful gas, helping the patients understanding the basic knowledge of COPD and common respiratory system drugs, understanding general and special therapies of COPD, learning skills to control disease condition such as abdominal respiration, pulmonary rehabilitation with pursed lips breathing and correct cough and expectoration, understanding monitoring of disease condition and correct time to go to hospital for visiting, and understanding respiratory rehabilitation exercise routines and rehabilitation assessment techniques.

Next was exercise training. Maximum motion power (W_max_) was detected through cardiopulmonary exercise test before rehabilitation. Lower limbs exercise prescription was formulated by respiratory disease specialists. Power bicycle endurance exercise was adopted to train lower limbs, 30 minutes each time, three times each week; NOMARK 828E power bicycle (NOMARK, Sweden) was used. Moreover oxygen inhalations via nasal tube at oxygen flow of 2~3 L/minutes was given to strengthen the exercise endurance capability of the patients. The initial target was low-strength exercise at 30%~40% W_max_. With the improvement of the capacity, the exercise strength was increased, and moderate-strength exercise at 50%~60% W_max_ was the target. Exercise continued after the target was achieved. The patients did respiration gymnastics, pursed lips breathing and abdominal respiration under the guidance of specialized rehabilitation therapists, 15 minutes each time, three times each week. Pursed lips breathing refers to inhaling via nostril and exhaling via pursed lips. The initial time ratio of inhalation to exhalation was 1:2, and time ratio of inhalation to exhalation at 1:4 was taken as the target. The third content was medication guidance. Clinical pharmacists provided consultation and guidance about internal medicines and inhaled drugs for patients. Last was social psychological support. Psychotherapists timely gave psychological guidance to COPD patients who were anxious and depressed and provide helps to the patients if they had difficulties.

### Observation indicators and evaluation methods

Patients in the three groups were evaluated before treatment and in the 3^rd^ month after treatment. Cardiopulmonary exercise testing (CPET) was performed using cardio-pulmonary function tester (Sendisi Company, USA). The power was increased to symptom limited maximum exercise load by a cycle ergometer. The patients warmed up at 0 W for 2 min. The treadmill load started from four weeks and gradually increased to 10~25 W/minutes. The rotating speed was kept between 40 r/min and 60 r/minutes. The power was increased until the patient felt difficulty in doing the exercise because of dyspnea and fatigue or the rotating speed became lower than 40 r/min. The test stopped when indicators such as heart rate recovered. The exercise tolerance index was recorded. The nutritional condition, degree of airflow obstruction, degree of dyspnea and exercise tolerance were evaluated using BODE indexes. The nutritional condition was expressed by body mass index (BMI). Degree of airflow obstruction was expressed by FEV1%. CHESTAC-8800D spirometer (CHEST, Japan) was used. The degree of dyspnea (Dyspnea-a) was scored using modified British Medical Research Council (MMRC). Exercise tolerance was expressed by 6MWD. FEV1%, MMRC and 6-WMD were given 0~3 points, and BMI was given 0~1 point; the total score was 10 points. Higher score indicated severer disease condition. Quality of life was scored using COPD-QOL scale edited by Fang et al.^9^ The scale included 4 factors and 35 items including factor 1~13 for ability of daily living, 14~20 factors for social activity, factor 21~28 for depression psychological symptom and factor 29~35 for anxiety. Every item was evaluated as 1~4 points, 1 point for the best and 4 points for the poorest. Every factor score was obtained by dividing the sum of the scores by number of the items. Arterial blood gas analysis was performed before treatment and in the 3^rd^ months after treatment. Femoral arterial blood was extracted when the patients were not supplied with oxygen and detected for PaO_2_ and PaCO_2_.

### Statistical Processing

Data were processed using SPSS ver. 21.0. Quantitative data were expressed in the form of mean ± standard deviation (SD). Pairwise comparison was performed using least significant difference. Games-Howell method when variance was not homogeneous. Difference was statistically significant if P<0.05.

## RESULTS

The differences of W_max_, VO_2max_, 6MWD, BMI, MMRC, FEV1% and BODE indexes between the three groups had no statistical significance (P>0.05). The W_max_, VO_2max_, 6MWD, MMRC and BODE indexes of intervention group 1 and 2 were significantly improved after treatment (P<0.01); and the differences of the above indexes between the three groups had statistical significance (P<0.05). The 6MWD and MMRC of intervention Group-B were superior to those of intervention group 1 (P<0.05). The differences of BMI and FEV1% between the three groups had no statistical significance before and after treatment (P>0.05). The W_max_, VO_2max_, 6MWD, BMI, MMRC, FEV1% and BODE indexes of the control group had no significant difference before and after treatment (P>0.05) (Table-II).

**Table-II:** Exercise tolerance and BODE indexes of the three groups before and after treatment.

Group	Control group		Intervention group 1		Intervention group 2	

	Before	After	Before	After	Before	After
W_max_(W)	69.83±12.02	68.41±10.52	68.53±14.40	75.37±12.48^ab^	69.74±14.06	77.63±12.86^ab^
VO_2max_(L/ min)	1.02±0.15	1.00±0.16	1.03±0.15	1.08±0.11^ab^	1.04±0.12	1.11±0.13^ab^
6MWD(M)	254.24±17.96	249.57±17.80	256.28±17.72	311.09±20.15^ab^	253.25±17.40	332.87±17.90^abc^
BMI(kg/m^2^)	21.45±1.62	21.13±1.79	21.30±1.72	21.49±1.44	21.24±1.50	21.37±1.46
MMRC(point)	2.85±0.34	2.86±0.41	2.85±0.36	2.51±0.52^ab^	2. 86±0.34	2.22±0.40^abc^
FEV1%	42.01±4.55	41.87±4.39	43.23±5.96	42.46±4.93	42.26±5.14	43.52±4.71
BODE indexes	5.86±1.11	5.89±0.92	5.85±0.81	4.45±0.57^ab^	5.86±0.75	4.34±0.47^ab^

***Note:***

a indicated P<0.05 compared before treatment;

b indicated P<0.05 compared to the control Group-After treatment;

c indicated P<0.05 compared to intervention group 1 after treatment.

The PaO_2_ and PaCO_2_ of the three groups had no significant differences before treatment (P>0.05), The PaO_2_ and PaCO_2_ of intervention Group-A and B were obviously improved after treatment, and the difference before and after treatment was statistically significant (P<0.05). The improvement of PaO_2_ and PaCO_2_ of intervention Group-B were superior to that of intervention Group-A, and the difference was statistically significant (P<0.05). But the PaO_2_ and PaCO_2_ of the control group had no obvious changes after treatment compared to before treatment (P>0.05) (Table-III).

**Table-III:** Arterial blood gas indexes of the three groups before and after treatment.

Group	Control group		Intervention group 1		Intervention group 2	

	Before	After	Before	After	Before	After
PaCO_2_ (mm Hg)	52.60±4.68	53.25±4.01	52.69±4.73	48.66±5.42^ab^	53.80±4.42	43.65±4.03^abc^
PaO_2_ (mm Hg)	65.79±5.37	64.46±6.23	65.56±5.11	70.18±4.16^ab^	66.13±5.18	75.06±6.25^abc^

***Note:***

a meant that P<0.05 compared to before treatment;

b meant that P<0.05 compared to the control Group-After treatment;

c meant that P<0.05 compared to intervention group 1 after treatment.

**Fig.1:**
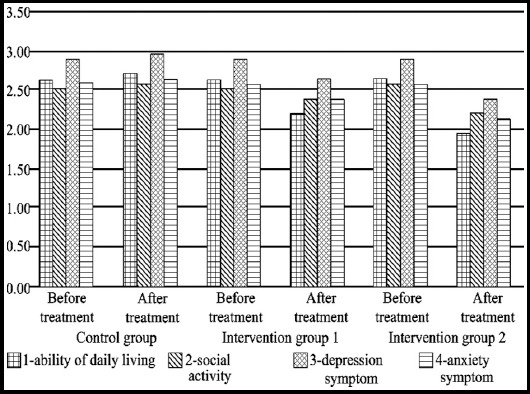
Relevant indexes of living quality of the three groups before and after treatment

There was no statistically significant difference in the score of living quality between the three groups before treatment (P>0.05). The ability of daily living, social activity, depression symptom and anxiety symptom of intervention group 1 and 2 were apparently improved after treatment, and the differences were statistically significant (P<0.05); the differences of those indexes between the two intervention groups and control group were also statistically significant after treatment (P<0.05). Moreover the ability of daily living, social activity, depression symptom and anxiety symptom of intervention group 2 had more obvious improvement compared to intervention group 1, and there were statistically significant differences (P<0.05). But no differences were observed in the relevant indexes of living quality of the control Group-Before and after treatment (P>0.05) (Fig.1).

## DISCUSSION

COPD is featured by high incidence and fatality rate. Most of COPD patients have symptoms of dyspnea, weakened exercise capability and malnutrition, which reduce their living quality.^10^ Therefore early effective treatment has great significance to the improvement of disease condition.

NPPV is an effective auxiliary therapy for patients with COPD at remission stage.^11^ NPPV can provide patients with positive end expiratory pressure, relieve respiratory muscle fatigue, increase alveolar ventilation volume, improve pulmonary function, and correct hyoxemia to reduce frequency of dyspnea and promote recovery.^12^ Application of NPPV during exercise can improve PaO_2_, reduce PaCO_2_, increase the possibility of attending exercise, improve exercise endurance, and extend exercise time.^13^ Long-term application of NPPV for patients with severe COPD can enhance living quality, relieve dyspnea, improve exercise endurance, reduce frequency of acute attack and treatment and hospitalization time, and decrease rate of tracheal intubation and fatality rate. It is especially effective for COPD patients with Gold grade III or IV.^14^ Duiverman et al.^15^ found that NPPV further could improve the respiratory rehabilitation effect of patients with severe COPD. The results of this study suggested that the improvement of exercise endurance, dyspnea, pulmonary function indicators and living quality score of the observation group was significantly superior to that of the control group, indicating that multidisciplinary comprehensive respiratory rehabilitation in combination with NPPV could further improve rehabilitation effect and had a special role in increasing partial pressure of blood oxygen, relieve CO_2_ retention, improving exercise endurance and relieve dyspnea.

Respiratory rehabilitation is suitable for patients with COPD in stable phase. Effective comprehensive respiratory rehabilitation is helpful to delay disease progress and reduce or prevent acute attack; hence it is an effective means to relieve dyspnea and improve exercise endurance and living quality.^16^,^17^ Pulmonary rehabilitation plan for COPD patients include assessment, exercise training, health education, nutritional interference and social psychosocial support, which involves multiple subjects. In recent years, multidisciplinary cooperation mode has been more and more recognized by the medical field. In 2004, American Thoracic Society and European Respiratory Society emphasized the important of respiratory rehabilitation exercise based on multidisciplinary cooperation.^18^ A study has pointed out that respiratory rehabilitation training based on multidisciplinary cooperation is quite effective in enhancing the living quality and exercise endurance of elders with moderate or severe COPD.^19^ Multidisciplinary cooperation was adopted in this study. The respiratory specialists evaluated disease condition, treated and educated patients, and formulated and adjusted rehabilitation plans. The respiratory nurses guided the patients to do respiratory exercise, pursed lips breathing, abdominal respiration, and lower limbs exercise. The clinical pharmacists answered the questions asked by the patients and puzzles of their family members. Nutritionists guided the patients to eat reasonably. The psychologists timely identified COPD patients with anxious and depressed psychology and gave them psychological counseling. The multi-disciplinary team cooperated to provide comprehensive medical services for the patients. The research results suggested that the 6-MWD, MMRC, PaO_2_, PaCO_2_ and score of quality of life of intervention Group-B were obviously superior to those of intervention Group-A, indicating that multidisciplinary comprehensive respiratory rehabilitation could significantly improve the clinical efficacy of comprehensive respiratory rehabilitation for COPD patients.

## Conclusion

Multidisciplinary comprehensive respiratory rehabilitation in combination with NIPPV has high clinical values in the rehabilitation process of COPD patients. Implementing reasonable and effective health education, rehabilitation guidance, medication guidance, nutritional guidance and psycho-health guidance can further strengthen the recognition of patients on COPD and improve compliance. Multidisciplinary comprehensive respiratory rehabilitation in combination with NIPPV can further relieve dyspnea and improve exercise endurance and living quality, which is worth promotion.

## References

[1] Du ST, Xing B, Ding LM, Wang CX, Yang FB, Liu ZL (2014). The combined effects of pulmonary rehabilitation and bronchial dilation drugs on patients with advanced pulmonary disease. Chin J Phys Med Rehab.

[2] Ries AL, Bauldoff GS, Carlin BW, Casaburi R, Emery CF, Mahler DA (2007). Pulmonary rehabilitation:joint ACCP/AACVPR evidence-based clinical practice guidelines. Chest.

[3] Wu HH, Gao FD, Zhan JJ (2015). Clinical efficacy of the treatment of tiotropium bromide combined salmeterol/fluticasone on chronic obstructive pulmonary disease. Chin J Clin Pharm.

[4] Chen YK, Sun JX, Guo HQ, Li SW, Zhang HQ (2011). Influence of Seretide intake on arterial blood gas and pulmonary function of medium and severe COPD patients. Chin Med Herald.

[5] Li J, Chen TF, Xu QS, Chen QZ (2015). Clinical observation of seretide inhalation treatment of 82 cases of medium and severe chronic obstructive pulmonary disease. J Gannan Med Coll.

[6] Zhu ML, Cui W, Wu CH, Huang Y, Wan Xia, Deng RL (2013). Study on the role of the multidisciplinary team in the course of rehabilitation and nursing for COPD patients from hospital to community. Chin Gener Pract.

[7] Rabe KF, Hurd S, Anzueto A, Barnes PJ, Buist SA, Calverley P (2007). Global strategy for the diagnosis, management and prevention of chronic obstructive pulmonary disease:GOLD executive summary. Am J Respir Crit Care Med.

[8] Chronic Obstructive Pulmonary Disease Committee, Respiratory Society, Chinese Medical Association (2013). Guidelines for the Diagnosis and Treatment of Chronic Obstructive Pulmonary Disease (2013). Chin J Tubercul Resp Dis.

[9] Fang ZJ, Cai YY, Wang LH, Liang YJ, Chen JR, Deng XQ (2001). Living quality evaluation form for COPD patients and its application. Mod Rehabil.

[10] Marquis K, Maltais F, Duguay V, ezeau AM, LeBlanc P, Jobin J, Poirier P (2016). The metabolic syndrome in patients with chronic obstructive pulmonary disease. Egypt. J. Chest Dis. Tuberc.

[11] Borghi-Silva A, Di Thommazo L, Pantoni CB, Mendes RG, Salvini Tde F, Costa D (2009). Non-invasive ventilation improves peripheral oxygen saturation and reduces fatigability of quadriceps in patients with COPD. Respirol.

[12] Celli BR, Mac Nee W, ATS/ERS Task Force (2004). Standards for the diagnosis and treatment of patients with COPD:a summary of the ATS / ERS position paper. Eur Respir J.

[13] Kolodziej MA, Jensen L, Rowe B, Sin D (2007). Systematic review of noninvasive positive pressure ventilation in severe stable COPD. Eur Respir J.

[14] Khnlein T, Schnheit-Kenn U, Winterkamp S, Welte T, Kenn K (2009). Noninvasive ventilation in pulmonary rehabilitation of COPD patients. Respir Med.

[15] Duiverman ML, Wempe JB, Bladder G, Vonk JM, Zijlstra JG, Kerstjens HA (2011). Two-year home-based nocturnal noninvasive ventilation added to rehabilitation in chronic obstructive pulmonary disease patients:a randomized controlled trial. Respir Res.

[16] Derom E, Marchand E, Troosters T (2007). Pulmonary rehabilitation in chronic obstructive pulmonary disease. Ann Readapt Med Phys.

[17] Tian YJ, Liu QG, Li JH, Zhao LL, Zhao SY, Zhou MZ (2017). Effect of Multidisciplinary Comprehensive Pulmonary Rehabilitation on Aged Patients with Pneumoconiosis. Chin J Rehabil Theory Pract.

[18] Clini E, Sturani C, Rossi A, Viaggi S, Corrado A, Donner CF (2002). The Italian multicentre study on noninvasive ventilation in chronic obstructive pulmonary disease patients. Eur Respir J.

[19] Baltzan MA, Kamel H, Alter A, Rotaple M, Wolkove N (2004). Pulmonary rehabilitation improves functional capacity in patients 80 years of age or older. Can Respir J.

